# Targeted and immunotherapy based on tissue of origin in carcinoma of unknown primary: a two-case report and literature review

**DOI:** 10.3389/fonc.2026.1741467

**Published:** 2026-03-03

**Authors:** Mei Wang, DongYue Yan, Wen Sun, Jingshan Liang

**Affiliations:** The Affiliated Lianyungang Hospital of Xuzhou Medical University, The First People’s Hospital of Lianyungang, Lianyungang, China

**Keywords:** cancer, carcinoma of unknown primary, case report, targeted and immunotherapy, test of tumor origin

## Abstract

**Background:**

Patients with carcinoma of unknown primary (CUP) generally have a poor prognosis due to the lack of effective treatment options resulting from unclear diagnosis. Determining the tumor type through tumor origin testing, followed by cancer-specific genetic testing and precision therapy, may potentially improve the prognosis of CUP patients.

**Case presentation:**

Case 1: A 44-year-old male patient presented to a local hospital with lower limb pain. A bone biopsy pathological report from our hospital indicated metastatic carcinoma in the bone lesion. The 90-gene expression analysis yielded a similarity score of 56.9, suggesting a high probability of lung cancer origin. Based on the genetic testing and combined with immunohistochemistry results, the diagnosis was metastatic adenocarcinoma. Subsequently, the bone biopsy tissue was tested for Epidermal growth factor receptor/Anaplastic Lymphoma Kinase/ROS proto-oncogene 1 (*EGFR/ALK/ROS1)* gene mutations, which revealed an *EGFR* exon 19 deletion (19Del) mutation. Based on the above results, the patient received chemotherapy with carboplatin and pemetrexed disodium combined with targeted therapy using the EGFR tyrosine kinase inhibitor (TKI) almonertinib mesylate tablets. Based on the molecular evidence provided by the tumor origin test results, a diagnosis was established for the patient by the clinician, and corresponding treatment plans were formulated accordingly. Unfortunately, after three cycles of treatment, the patient discontinued therapy due to other issues and was lost to follow-up. Case 2: A 59-year-old male patient sought medical attention in May 2021 due to dysphagia. He underwent radical esophagectomy for esophageal cancer at an external hospital, with postoperative pathological diagnosis of esophageal squamous cell carcinoma. In August 2024, he presented with cervical lymph node enlargement. A biopsy pathological diagnosis was metastatic poorly differentiated carcinoma, with current markers showing no definitive differentiation towards adenocarcinoma or non-keratinizing squamous cell carcinoma. The gene expression profile results indicated that the tumor sample was most likely derived from gastric and esophageal tissues, i.e., highly suggestive of gastric/esophageal cancer, with a similarity score of 96.4. Based on the patient’s medical history and immunohistochemistry results, the clinicians considered a diagnosis of esophageal squamous cell carcinoma (with lymph node metastasis). According to this diagnosis, the patient received six cycles of immunotherapy combined with chemotherapy. Regular follow-up examinations showed gradual shrinkage of the lymph nodes. A re-examination on August 12, 2025, indicated stable disease, with a progression-free survival (PFS) already reaching twelve months.

**Conclusion:**

The two cases reported in this paper demonstrate that targeted and immune treatment plans based on the tissue of origin of the tumor can serve as a clinical option for patients with CUP. These findings may provide new information and references for clinical decision-making in the management of CUP.

## Introduction

Carcinoma of unknown primary (CUP) refers to a malignancy for which the primary site cannot be identified by conventional diagnostic methods. Based on clinicopathological characteristics, CUP patients can be categorized into two prognostic subgroups. The first subgroup is the favorable-prognosis subgroup (15%–20%) (1), whose clinical and pathological features strongly suggest a specific tissue of origin. This subgroup includes patients with: neuroendocrine carcinoma of unknown primary, peritoneal carcinomatosis of serous papillary subtype, women with isolated axillary lymph node metastases, squamous cell carcinoma involving non-supraclavicular cervical nodes, a single metastatic site of unknown primary, and male patients with osteoblastic bone metastases and prostate-specific antigen (PSA) expression. Tumors in the favorable-prognosis subgroup are typically chemosensitive and demonstrate better outcomes when treated with site-specific therapy. The second subgroup is the unfavorable-prognosis subgroup (80%–85%) (2). Due to the inability to identify the primary tumor, this subgroup is usually managed with empiric chemotherapy and has a poor prognosis. With further research into CUP, more favorable-prognosis subtypes within the unfavorable-prognosis subgroup have been identified, including colorectal-type, lung-type, and renal-type carcinomas of unknown primary (3). These subtypes correspond to specific treatment strategies and are associated with improved survival rates. Therefore, one of the major challenges currently is how to better identify the characteristics of more primary tumors within the unfavorable-prognosis subgroup to enable targeted therapy for these patients. Advances in molecular biology have facilitated classification based on epigenetic alterations in different tumors (4), which aids in implementing optimal individualized treatment for CUP patients and is of significant importance for improving therapeutic outcomes.

Herein, we present two cases in which the primary tumor site remained unidentified despite comprehensive clinical, imaging, pathological, and laboratory investigations. Tumor tissue origin detection based on gene expression profiling proposed potential hypotheses regarding the tissue of origin. Subsequent tumor-specific genetic testing and the observed clinical treatment response provided further corroboration for these findings. The possible tumor origins suggested by the tissue-of-origin assay offered valuable reference for clinicians to adjust the patients’ treatment strategies. This facilitated a shift from traditional broad-spectrum chemotherapy to more targeted approaches, including lung adenocarcinoma-specific chemotherapy combined with targeted therapy, and esophageal carcinoma chemotherapy combined with immunotherapy. Consequently, the patients with CUP received more precise treatment, which may potentially improve their prognosis. This holds significant clinical importance for CUP patients.

## Cases report

### Case 1

**Figure 1:**
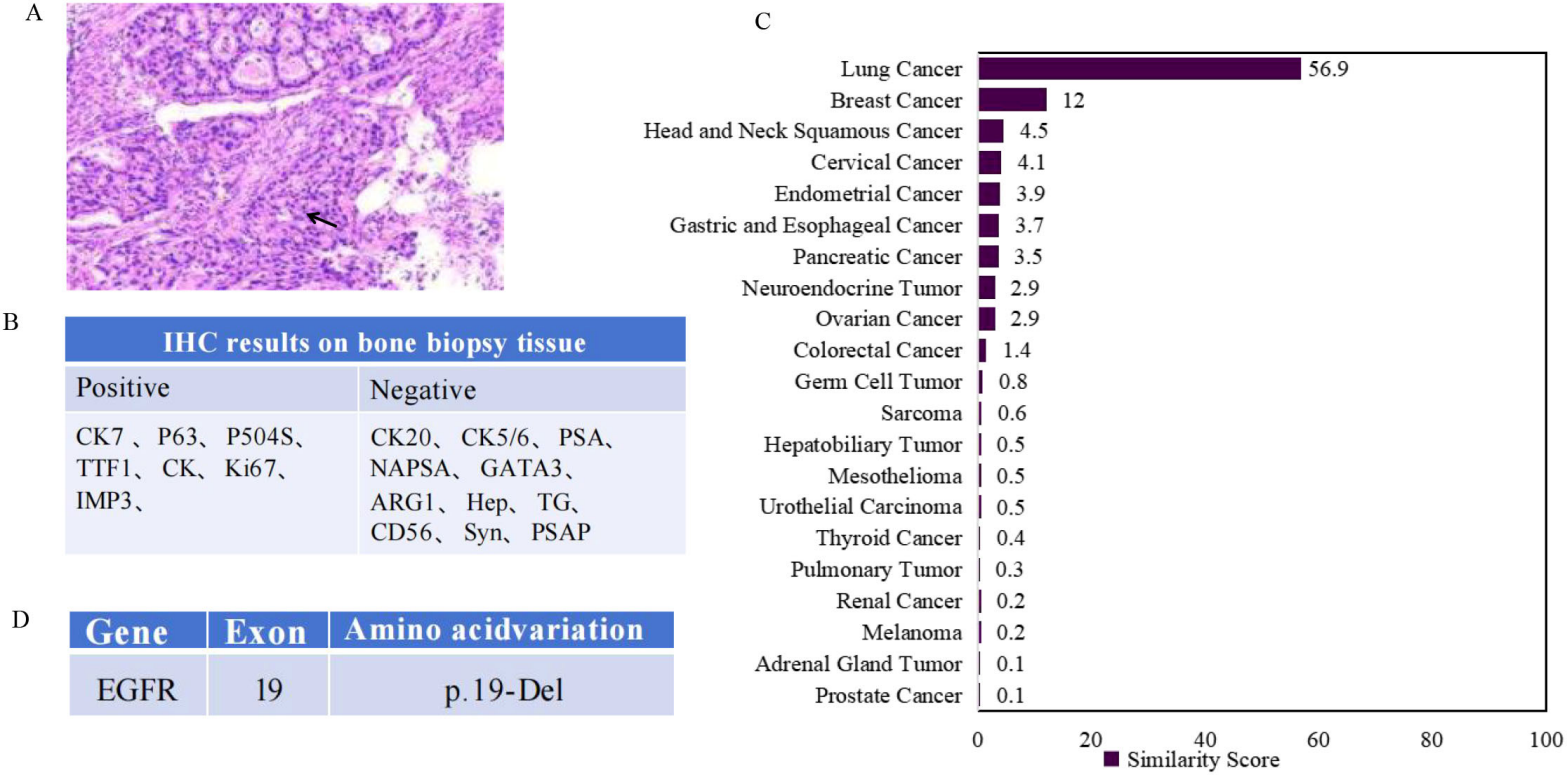
Summary of immunohistochemical and molecular testing results. **(A)** Hematoxylin and eosin staining on bone mass showed metastatic carcinoma. **(B)** Pathological and immunohistochemical (IHC) results. **(C)** 90-gene tumor tissue traceability expression assay results. The maximum similarity score for tumor tissue traceability was 100. **(D)** Lung adenocarcinoma related gene mutations results of tissue specimens analyzed by PCR. EGFR, epidermal growth factor receptor.

**Figure 2:**
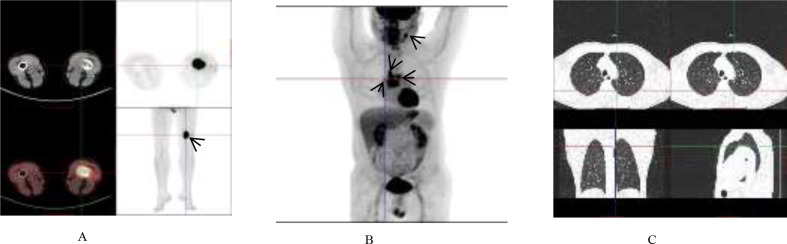
Summary of case l PET/CT images. **(A)** Metastatic lesion in the distal left femur. **(B)** Metastatic lesions in the mediastinal lymph nodes (regions 2R, 4R, and 4L) and the left para-carotid lymph node; **(C)** No significant increase in radiotracer uptake was observed in either lung.

A 44-year-old male presented to a local hospital on April 16, 2024, complaining of lower limb pain. Examination revealed a radiodense shadow in the distal left femur. On April 17, a pathological examination of a bone lesion biopsy performed at our hospital indicated metastatic carcinoma (**Figures 1A, B**). Based on immunohistochemical markers, further investigation of the lungs, head, and neck was recommended. To evaluate the systemic tumor burden, a positron emission tomography-computed tomography (PET-CT) scan was performed on April 26 (**Figures 2A-C**). The results showed focal wall thickening with increased glucose metabolism in the mid-esophagus, suggestive of esophageal carcinoma, and recommended gastroscopy. Destructive bone lesions with surrounding soft tissue masses in the distal left femur were considered metastatic. Enlarged mediastinal lymph nodes (regions 2R, 4R, and 4L) with increased glucose metabolism were also deemed metastatic. Enlarged lymph nodes adjacent to the left carotid artery with increased glucose metabolism could not rule out metastasis. No significant increase in radiotracer uptake was observed in either lung. No other abnormal foci of significantly increased glucose metabolism indicative of malignancy were detected in the rest of the body or the brain. To further investigate potential esophageal lesions, a painless electronic upper gastrointestinal endoscopy was performed on May 10, which revealed no esophageal abnormalities. Despite extensive evaluation using conventional computed tomography (CT) of the upper abdomen, mid-abdomen, and pelvis, thyroid ultrasonography, magnetic resonance imaging (MRI) of the left thigh and nasopharynx, whole-body PET-CT, whole-body bone scintigraphy, and immunohistochemistry, a definitive diagnosis could not be established. Due to the unidentified primary origin, and the patient was subjected to a broad-spectrum anticancer regimen incorporating carboplatin and paclitaxel with family consent, which also covers esophageal cancer. Carboplatin is a platinum-based chemotherapeutic agent that induces apoptosis by forming cross-links with tumor cell DNA, thereby interfering with its replication and transcription. Pemetrexed is an antifolate metabolic drug that inhibits tumor proliferation by blocking DNA synthesis through the suppression of key enzymes such as thymidylate synthase.

To help identify the tumor origin, tumor tissue origin testing was performed using the bone biopsy sample. On May 16, a 90-gene expression analysis yielded a similarity score of 56.9 (**Figure 1C**), indicating a high probability of lung origin. Based on the immunohistochemical findings, the diagnosis was considered to be metastatic adenocarcinoma, consistent with a pulmonary origin. Following the diagnostic and therapeutic guidelines for lung adenocarcinoma, the bone biopsy tissue was subsequently analyzed for driver gene mutations Epidermal growth factor receptor/Anaplastic Lymphoma Kinase/ROS proto-oncogene 1 (*EGFR/ALK/ROS1*) using fluorescent quantitative PCR. The test identified an *EGFR* exon 19 deletion (19Del) mutation (**Figure 1D**). Based on the comprehensive diagnostic and genetic findings, the patient received chemotherapy with carboplatin and pemetrexed disodium, combined with targeted therapy using the EGFR tyrosine kinase inhibitor (TKI) almonertinib mesylate. Aumolertinib mesylate, a third-generation EGFR-TKI, irreversibly binds to *EGFR* mutants, inhibiting downstream signaling pathways and thereby blocking tumor growth and metastasis. Thus, the patient obtained a relatively definitive diagnosis and received corresponding treatment. Regrettably, the patient discontinued therapy and was lost to follow-up due to other issues after three cycles of treatment, preventing assessment of the treatment efficacy.

### Case 2

**Figure 3:**
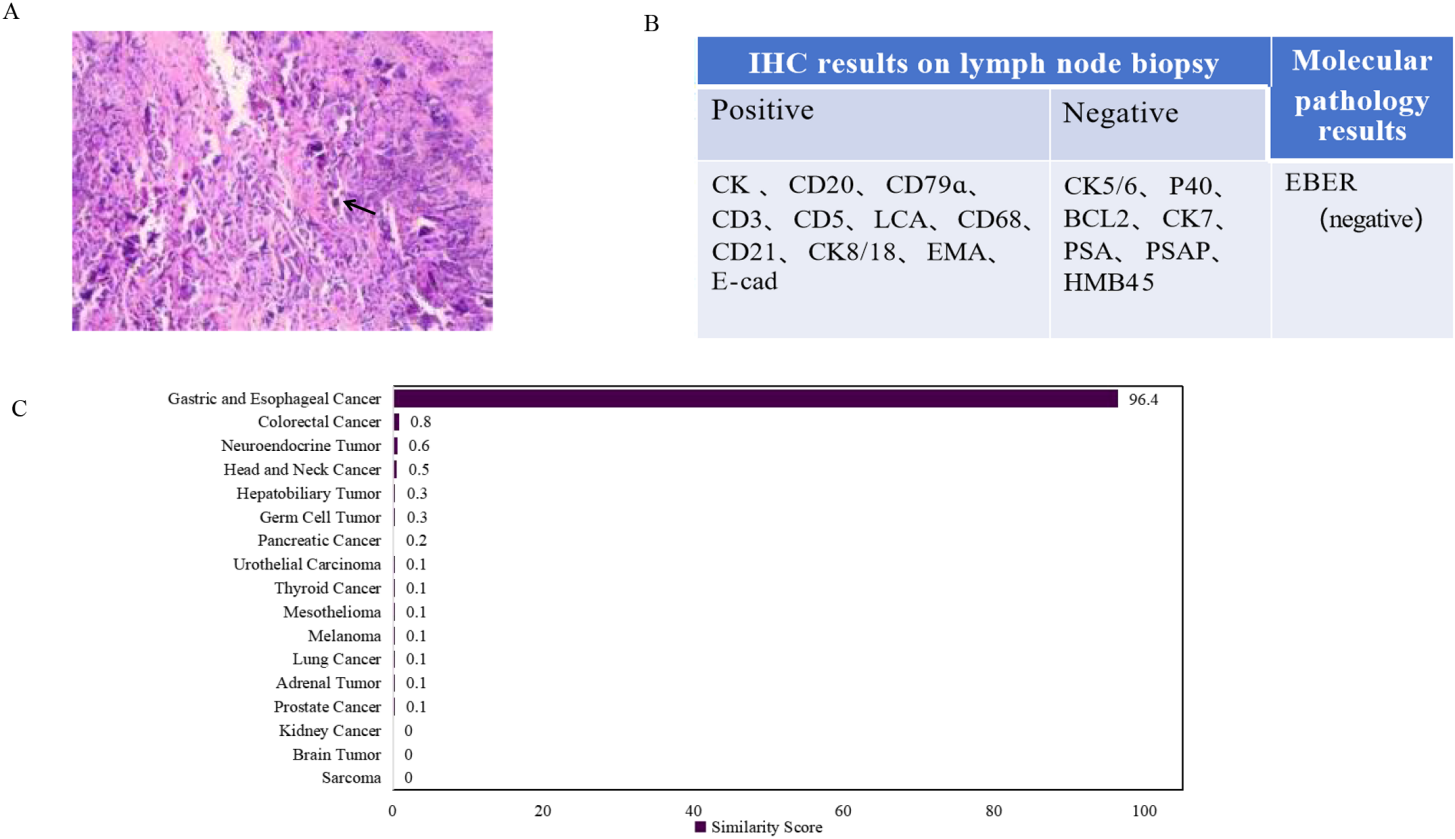
Summary of immunohistochemical and molecular testing results. **(A)** Hematoxylin and eosin staining on lymph node indicated metastatic poorly differentiated cancer. **(B)** IHC and molecular pathology results. **(C)** 90-gene tumor tissue traceability expressi on assay results. The maximum similarity score for tumor tissue traceability was 100.

A 59-year-old male patient presented with dysphagia in May 2021 and underwent radical esophagectomy for esophageal cancer at an external hospital. Postoperative pathological diagnosis confirmed esophageal squamous cell carcinoma. The patient subsequently received four cycles of chemotherapy at our hospital starting on August 9, 2021, with regular follow-up examinations indicating stable disease. On August 22, 2024, he presented with cervical lymph node enlargement. A biopsy revealed metastatic poorly differentiated carcinoma, with current markers showing no definitive evidence of adenocarcinoma or non-keratinizing squamous cell carcinoma differentiation (**Figures 3A, B**). Contrast-enhanced CT of the chest and entire abdomen on August 23 showed newly enlarged lymph nodes in the left supraclavicular region and mediastinum, suggestive of metastasis. A whole-body PET-CT scan on September 2 revealed post-esophagectomy status, with enlarged lymph nodes exhibiting increased glucose metabolism in the left supraclavicular region and mediastinal zone 2L, consistent with metastasis; no other abnormal hypermetabolic foci indicative of malignancy were detected in the rest of the body or the brain. Given the aforementioned atypical features and previous treatment history, to clarify the therapeutic direction, clinicians requested molecular testing for tumor tissue origin for the patient. The gene expression profile results indicated that the tumor sample was most likely derived from gastric and esophageal tissues, suggesting a high probability of gastric and esophageal carcinoma, with a similarity score of 96.4 (**Figure 3C**). Based on the medical history and immunohistochemistry results, the clinicians considered a definitive diagnosis of esophageal squamous cell carcinoma (lymph node metastasis). Based on this diagnosis, the patient received six cycles of immunotherapy (tislelizumab/adebrelimab) combined with chemotherapy (nab-paclitaxel and carboplatin). Chemotherapy (nab-paclitaxel and carboplatin) can eliminate tumor cells while potentially exposing more tumor antigens, analogous to “unmasking the enemy.” Concurrently, immunotherapy (tislelizumab/adebrelimab) functions to “release restrictions and activate our own forces (T cells),” enabling a more sustained and effective attack against these targets. This combination results in a synergistic therapeutic effect.

**Figure 4:**
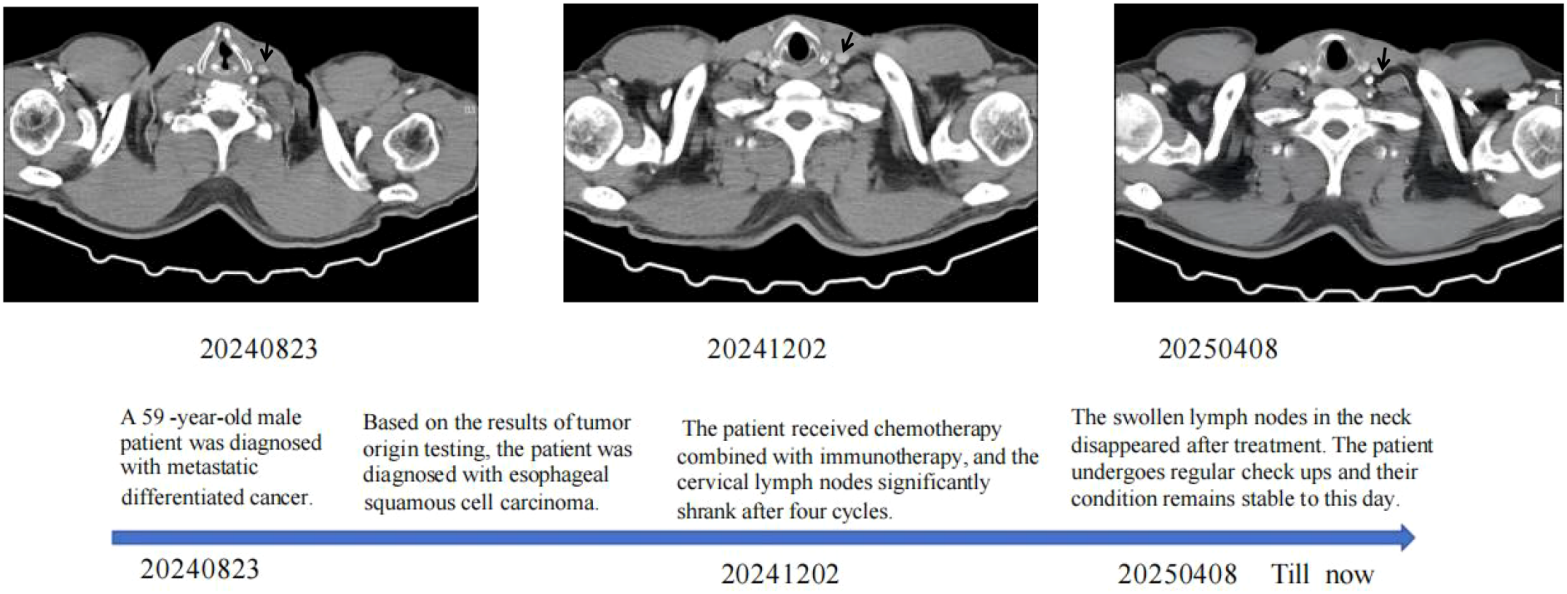
The patient's (case 2) clinical course schedule. Computed tomography scans of chest lesions before treatment (20240823), under treatment (20241202), and after treatment (2025 04 08). The patient got the treatment with immunotherapy (Tislelizumab/Adebrelimab) combined with chemotherapy (nab-paclitaxel and carboplatin). The patient undergoes regular follow-up examinations and has achieved a PFS of 12 months so far.

Follow-up enhanced CT scans after discharge demonstrated gradual shrinkage and eventual disappearance of the enlarged supraclavicular lymph nodes, as well as reduction in the size of the mediastinal lymph nodes, indicating a favorable treatment response. After discharge, the patient was maintained on oral Tegafur, Gimeracil, and Oteracil Potassium (S-1) with regular follow-up. As of the last follow-up on August 12, 2025, the patient’s condition remained stable, with a progression-free survival (PFS) of twelve months (**Figure 4**).

## Discussion

We present two relatively typical cases of CUP. Following multiple imaging and pathological examinations, and with the assistance of tumor tissue-of-origin gene testing, definitive diagnoses were established, leading to tumor-specific genetic testing and treatment. In Case 1, after a diagnosis of lung adenocarcinoma, genetic testing revealed an *EGFR* 19Del mutation. This finding, on the one hand, increased the likelihood of the patient benefiting from targeted therapy; on the other hand, it provided confirmatory evidence for the results of the tumor tissue-of-origin test. In Case 2, following lymph node metastasis, a diagnosis of esophageal squamous cell carcinoma was established, and the patient received chemotherapy combined with immunotherapy. The PFS has now reached 12 months, indicating a favorable therapeutic outcome. This result, on the one hand, demonstrates the efficacy of chemotherapy combined with immunotherapy in CUP patients; on the other hand, it also provides confirmatory evidence for the results of the tumor tissue-of-origin test.

The tumor tissue origin gene detection kit employed in this case is an ex vivo diagnostic method based on quantitative fluorescent PCR of tumor mRNA combined with artificial intelligence. By detecting and analyzing the expression of 90 signature genes in paraffin-embedded tumor samples, it calculates a similarity score to determine 21 common tumor types (5) (**Table 1**). When the similarity score is ≥45.3, the submitted tumor sample is most likely derived from that specific tumor type. Multiple retrospective studies have evaluated the overall accuracy of this product in CUP as ranging from 92.0% to 97.4% (6–9). Real-world studies indicate that this assay can support the provision of cancer type-specific treatment for 82.3% (116/141) of patients (5). A prospective randomized controlled clinical trial, the first of its kind globally focusing on CUP, provided the first international evidence for the efficacy of “site-specific therapy” in CUP. Studies have shown that patients with CUP whose tumor tissue of origin is predicted via genetic testing and who subsequently receive “site-specific therapy” achieve a median progression-free survival of 9.6 months. This is significantly superior to the 6.6 months observed with “traditional empirical chemotherapy” regimens, representing a 32% reduction in the risk of disease progression and a 9-month extension in median overall survival, thereby effectively improving the prognosis for this patient subgroup (10). However, according to the literature, current studies comparing site-specific therapy with empirical chemotherapy exhibit significant limitations. These include issues with patient recruitment (over-inclusion of chemotherapy-resistant tumor types and prolonged recruitment periods), limitations in study design (observational studies and trials with inherent flaws), heterogeneity in CUP classification methods (e.g., epigenetic analysis versus transcriptomic analysis), and non-comparability of treatment plans (11). Consequently, the inferences drawn from many studies are constrained. An evaluation of recently published literature on CUP has proposed the adoption of two comprehensive clinical trial design schemes: one prospective and one pragmatic. Both are suitable for incorporating the latest diagnostic and therapeutic advances to enhance the quality of CUP research and improve outcomes for a greater number of patients (12). It is believed that with the continuous advancement of CUP research, the classification and treatment strategies for CUP will become increasingly well-defined.

**Table 1:** List of 90 genes included in the tumor origin gene detection panel.

*ACPP*	*CDH1*	*CYP17A1*	*GPM6B*	*KLK3*	*MMP12*	*PIGR*	*S100P*	*SST*
*ACTG2*	*CDH17*	*DLK1*	*GPX3*	*KRT13*	*MMP3*	*PLA2G2A*	*SCGB2A2*	*SULT2A1*
*AGR2*	*CEACAM5*	*EPCAM*	*GREM1*	*KRT14*	*MSMB*	*POSTN*	*SERPINA3*	*TACSTD2*
*APOBEC3B*	*CEACAM6*	*ESR1*	*HBB*	*KRT15*	*NKX3-1*	*PRRX1*	*SERPINB3*	*TG*
*APOD*	*CHGA*	*FABP1*	*ID4*	*KRT19*	*NPTX2*	*PTGDS*	*SFN*	*TH*
*ASPN*	*CHI3L1*	*FABP4*	*IGFBP2*	*KRT20*	*NPY1R*	*PTN*	*SFRP1*	*TM4SF4*
*ATP1B1*	*CLDN18*	*GATA3*	*IGFBP7*	*LGALS4*	*PCDH7*	*RPS11*	*SFTPB*	*TSPAN8*
*AZGP1*	*CLU*	*GCG*	*IGJ*	*LUM*	*PCP4*	*RPS4Y1*	*SLC3A1*	*TYRP1*
*C7*	*COL11A1*	*GFAP*	*ISL1*	*MGP*	*PEG3*	*S100A2*	*SPINK1*	*VEGFA*
*CA12*	*CXCL14*	*GJA1*	*KLK2*	*MMP1*	*PI15*	*S100A8*	*SPP1*	*XIST*

With the continuous advancement of precision medicine, targeted therapies based on driver gene mutations have been incorporated into the standard treatment plans for certain primary tumors. For example, EGFR-TKIs treatment has become the first-line therapy for lung adenocarcinoma patients harboring *EGFR* mutations (13). However, whether relevant driver gene mutations characteristic of known primary tumors persist in CUP, the feasibility of administering targeted therapy, and its efficacy in this context require extensive clinical investigation. Literature reports indicate the presence of driver gene mutations in CUP, such as tumor protein p53 (TP53), Kirsten rat sarcoma viral oncogene homolog (*KRAS*) mutations (14–16), as well as alterations in *ALK*, *EGFR*, *RET*, *FGFR1*, and *NTRK1*. Patients harboring these mutations may potentially benefit from targeted therapies (17). Among six CUP cases with *EGFR* mutations reported in the literature who received EGFR-TKIs, four patients achieved a PFS ranging from six to eleven months (17–19). Therefore, it is reasonable to hypothesize that the patient in Case 1 would also likely benefit from lung adenocarcinoma-specific chemotherapy combined with EGFR-TKIs. Nonetheless, due to the limited number of reported cases, the benefit of targeted therapy for CUP patients remains unclear and warrants further validation through clinical practice. Importantly, while driver mutations are significant, evidence suggests their implications are tumor type-specific. Consequently, their value in CUP, where the tumor origin is unknown, remains uncertain. For example, the *BRAF* V600 mutation can occur in melanoma, colorectal cancer, non-small cell lung cancer, and papillary thyroid carcinoma. However, BRAF inhibitors are effective in melanoma and advanced papillary thyroid cancer patients with *BRAF* V600 mutations but are inactive in colorectal cancer and non-small cell lung cancer patients harboring the same mutation (20). This also represents a potential diagnostic approach for CUP highlighted in this report: First, clarify the tumor origin, and then perform tumor-specific genetic testing. However, whether this approach is applicable in clinical practice still requires extensive clinical research for exploration.

Immunotherapy represents a profound treatment modality for malignant tumors, demonstrating significant efficacy either as monotherapy or in combination with other chemotherapeutic agents. The use of immune checkpoint inhibitors (ICIs) has markedly improved survival rates in various malignancies (21–23). Studies have indicated that patients with CUP may be particularly sensitive to ICIs (24). In CUP, 28% of patients harbor one or more predictive biomarkers for ICIs response. Specifically, these include: programmed death-ligand 1 (PD-L1) expression ≥5% on tumor cells in 22.5% of patients (with 34% showing expression ≥1%), positive PD-L1 expression on lymphocytes in 58.7% of patients, microsatellite instability-high (MSI-H) status in 1.8% of patients, and a tumor mutational burden (TMB) ≥17 mutations per megabase in 11.8% of patients (25). Analysis suggests that a tumor mutational burden >10 mutations per megabase may provide a potential genomic correlate for ICIs response in CUP patients (26). However, these biomarkers require further validation in the CUP patient population. However, it is crucial to acknowledge that CUP patients often cannot access newer and more effective treatments (such as immunotherapy or molecular targeted therapy) because the approvals for most ICIs and targeted agents are disease-specific (27). Therefore, determining the most likely site of origin for CUP patients is urgently needed, as it facilitates the selection of optimal treatment plans and may improve prognosis and survival rates. In this case, the patient with lymph node metastasis was considered to have esophageal squamous cell carcinoma based on tumor origin testing combined with medical history. After treatment with immunotherapy combined with chemotherapy, the patient’s condition remained stable, and PFS has currently reached twelve months. Two reported cases of CUP achieved complete remission after receiving immunotherapy combined with chemotherapy, indicating favorable therapeutic outcomes (28, 29). Immunotherapy combined with chemotherapy may have a good therapeutic effect on CUP patients, but further extensive clinical practice is still required for exploration.

Regrettably, the patient in Case 1 did not persist with the treatment, and the underlying reasons warrant deep consideration. CUP patients often experience significant psychological and financial stress due to the prolonged diagnostic process, where conventional examinations (e.g., ultrasonography, MRI, CT, PET-CT) fail to provide definitive results and traditional pathology cannot yield a clear diagnosis, leading to anxiety about the disease diagnosis and prognosis. Therefore, an efficient and conclusive diagnostic workflow is crucial for CUP patients. An earlier definitive diagnosis enables the timely initiation of targeted therapy. As healthcare professionals facing complex diagnostic scenarios like CUP, in addition to providing adequate communication to alleviate patients’ psychological distress, it is more important to fully utilize professional diagnostic procedures to help patients obtain a definitive diagnosis promptly. This approach maximizes the potential for patients to receive more specialized treatment and achieve a better prognosis. The diagnostic approach presented in this report may provide valuable reference for the diagnosis of patients with CUP.

## Conclusion

This report presents the diagnostic and therapeutic processes of two patients with CUP. Based on the results of tumor origin detection, Case 1 underwent lung adenocarcinoma-specific genetic testing, leading to a shift from a broad-spectrum anticancer chemotherapy regimen to tumor-specific chemotherapy combined with precision targeted therapy. Case 2, who was subjected to immunotherapy combined with chemotherapy, achieved a favorable therapeutic response. A primary limitation of this report is that the patient in Case 1 was lost to follow-up after discontinuing treatment, precluding the acquisition of prognostic data under targeted therapy. However, the main objective of this report is to elucidate the critical role of tumor origin tracing and subsequent tumor-specific genetic testing and personalized treatment in the diagnosis and management of CUP, which has been adequately demonstrated.

## Data Availability

The original contributions presented in the study are included in the article/supplementary material. Further inquiries can be directed to the corresponding author.
